# A Rare Prolactin-secreting Pituitary Carcinoma With Epidural and Thecal Metastases

**DOI:** 10.1210/jcemcr/luae047

**Published:** 2024-04-24

**Authors:** Anna Liu, Stan Van Uum, Donald Lee, Robert R Hammond, Shereen Ezzat, Kristin K Clemens

**Affiliations:** Schulich School of Medicine and Dentistry, Western University, London, ON N6A 3K7, Canada; Center for Diabetes, Endocrinology and Metabolism, St. Joseph's Health Care London, London, ON N6A 4V2, Canada; Department of Medicine, Division of Endocrinology and Metabolism, Western University, London, ON N6A 5W9, Canada; Department of Medical Imaging, Western University, London, ON N6A 5A5, Canada; Department of Pathology and Laboratory Medicine and Department of Clinical Neurological Sciences, Western University, London, ON N6A 5A5, Canada; Endocrine Oncology Site Group, Princess Margaret Cancer Centre, Toronto, ON M5G 2M9, Canada; Center for Diabetes, Endocrinology and Metabolism, St. Joseph's Health Care London, London, ON N6A 4V2, Canada; Department of Medicine, Division of Endocrinology and Metabolism, Western University, London, ON N6A 5W9, Canada; Department of Epidemiology and Biostatistics, Western University, London, ON N6G 2M1, Canada; Lawson Health Research Institute, London, ON N6C 2R5, Canada

**Keywords:** pituitary carcinoma, prolactin-secreting, metastases, pituitary tumor, case report

## Abstract

Pituitary carcinomas are rare but associated with significant morbidity and mortality. They remain challenging to diagnose and manage. In this case, we describe a 56-year-old man who presented with erectile dysfunction and binocular vertical diplopia. He had central hypogonadism, secondary adrenal insufficiency, and central hypothyroidism on biochemical testing. His serum prolactin was 1517 mcg/L (1517 ng/mL; reference range 4-15 mcg/L), and his sellar magnetic resonance imaging showed a 2.0 × 2.2 × 3.1 cm pituitary tumor. Pathology revealed a prolactin-secreting carcinoma. Despite treatment with a high-dose dopaminergic, 2 transsphenoidal resections, and 1 course of radiation, prolactin levels continued to rise. He developed metastases to the epidural space and thecal sac from the thoracic to sacral spine, for which he received 12 cycles of temozolomide chemotherapy with initial clinical and biochemical response. This was followed by disease escape and progression. We discuss the clinical and imaging features that warrant a high index of suspicion for pituitary carcinoma and review contemporary treatment.

## Introduction

Pituitary carcinomas are rare, comprising only .4% of all pituitary tumors (1). However, patients with carcinoma face a poor prognosis with fewer than 50% surviving beyond a year of being diagnosed with metastasis (2). We present a case of a prolactin-secreting pituitary carcinoma, discuss its diagnostic challenges, and provide insight into the signs and symptoms that should prompt a higher index of suspicion for this condition.

## Case Presentation

A 56-year-old man, with a history of well-controlled type 2 diabetes, presented with erectile dysfunction and double vision. On physical exam, he had binocular vertical diplopia.

## Diagnostic Assessment

**Figure 1.**
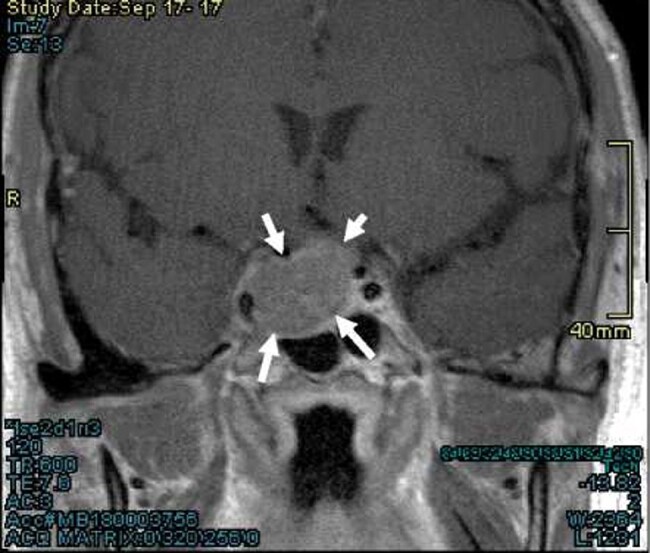
Coronal T1 view of magnetic resonance imaging sella at initial presentation showing a 2.0 × 2.2 × 3.1 cm central lesion with extension to the right cavernous sinus and suprasellar cistern reaching the optic chiasm.

Biochemical evaluation revealed an elevated serum prolactin of 1517 mcg/L (reference range 4-15 mcg/L) (1517 ng/mL), testosterone of .2 nmol/L (reference range 8.4-28.8 nmol/L) (5.8 ng/dL), luteinizing hormone of <1 IU/L (reference range 2-16 IU/L), and an inappropriately normal follicle stimulating hormone of 2.9 IU/L (reference range 1.3-19.3 IU/L). He had a low morning cortisol of 45 nmol/L (16.3 ng/mL; reference range 133-537 nmol/L), adrenocorticotropic hormone of 3.7 pmol/L (16.8 pg/mL; reference range <14 pmol/L), free thyroxine of 4.8 pmol/L (3.7 pg/mL; reference range 7.2-21 pmol/L), and a TSH of 1.6 mIU/L (reference range .3-5.6 mIU/L). Magnetic resonance imaging (MRI) of his sella revealed a 2.0 × 2.2 × 3.1 cm pituitary mass (Fig. 1). He was diagnosed with a pituitary macroprolactinoma with central hypogonadism, secondary adrenal insufficiency, and central hypothyroidism.

## Treatment

**Figure 2.**
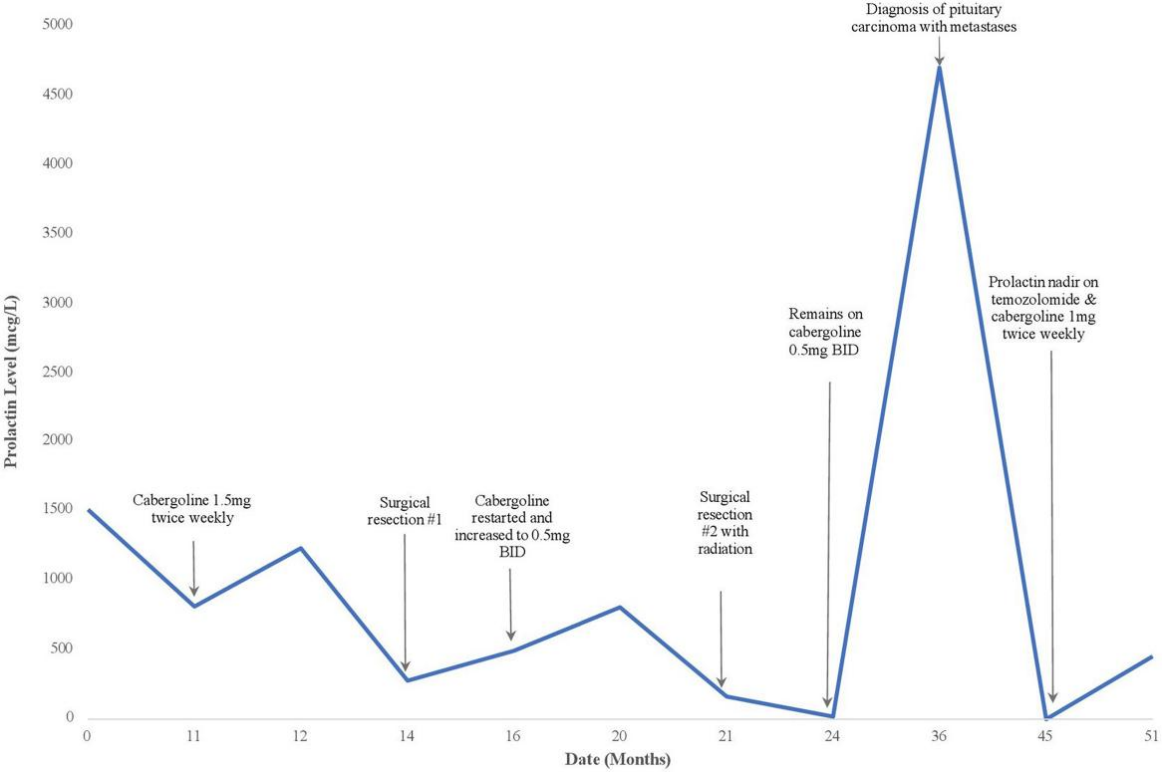
Prolactin levels over the course of diagnosis and treatment.

The patient was then started on cabergoline, which was titrated up to 1.5 mg twice weekly. He was also prescribed testosterone gel 7.5 g daily, levothyroxine 150 mcg daily, and hydrocortisone 10 mg before breakfast and 5 mg before supper. His serum prolactin level initially declined to 820 mcg/L (820 ng/mL) but 1 year later rebounded to 1239 mcg/L (1239 ng/mL) despite full adherence with therapy (Fig. 2).

**Figure 3.**
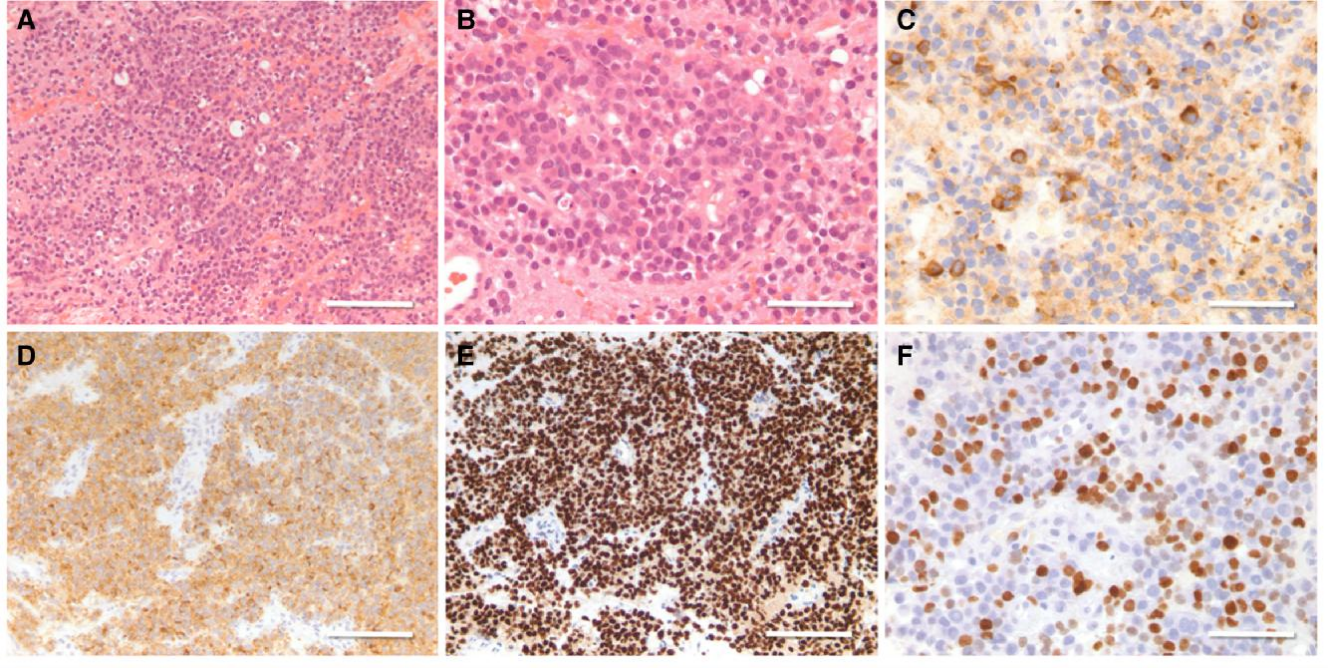
(A) The normal adenohypophyseal acinar architecture is replaced by a vaguely lobular growth pattern, H&E, bar = 100 µm. B) Individual cells reveal pale granular cytoplasm, mildly pleomorphic nuclei, and sparse mitotic figures (H&E, bar = 50 µm) (C) Chromogranin is variably abundant (antichromogranin immunoperoxidase, bar = 50µm). (D) Prolactin is expressed in tumor cell cytoplasm (antiprolactin immunoperoxidase, bar = 100 µm). (E) Tumor cells express nuclear Pit-1 transcription factor (anti-Pit-1 immunoperoxidase, bar = 100 µm). (F) The Ki67 index averages 25% (anti-Ki67 immunoperoxidase, bar = 50 µm). Abbreviation: H&E, hematoxylin and eosin.

The rising prolactin level along with worsening visual symptoms prompted endoscopic transsphenoidal resection. Pathology confirmed a sparsely granulated prolactin-secreting tumor with a high Ki-67 proliferation index of 20% to 25% (Fig. 3). Less than 10% of cells expressed nuclear methylguanine-DNA methyltransferase (MGMT) protein (low). D2 receptor expression was not available for testing.

**Figure 4.**
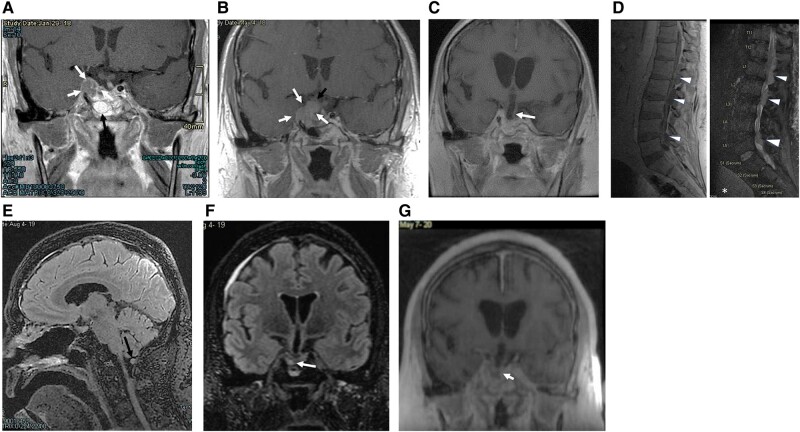
(A) Post gadolinium T1 coronal MRI following first resection in 2017. Recurrent tumor highlighted with white arrows. Enhancing mucosa in the sphenoid sinus (black arrow). (B) Post gadolinium T1 coronal MRI prior to second surgery in 2018. Enlargement of tumor (white arrows) encroaching on chiasm and right optic nerve (black arrow). (C) Post gadolinium T1 coronal MRI after second resection in 2018. Significant tumor debulking; interval lateral and third ventricular enlargement with enlarged third ventricle extending into sella (white arrow) (D) T1 sagittal (3A) and FSEIR sagittal (3B) MRI at metastases of prolactin-secreting pituitary carcinoma showing multiple epidural masses in lumbar spine at L1, L2-3, and L4-5 with dural compression. Note enlarged bladder due to cauda equina compression (asterisk). (E and F) At diagnosis of pituitary carcinoma in August 2019. FLAIR sagittal and coronal. No significant change in right-sided intrasellar tumor (white arrow). More bulk of left-sided pituitary tissue. Metastatic nodule inferior to cerebellar tonsil (black arrow). (G) Post gadolinium T1 coronal at prolactin nadir in May 2020. Tumor residual remains small (white arrow). Abbreviation: MRI, magnetic resonance imaging.

Postoperative prolactin decreased to 286 mcg/L (286 ng/mL) with residual tumor in the sella measuring .9 × .8 × .5 cm and right cavernous sinus measuring 2 × 1.4 × 1.8 cm (Fig. 4A). Two months after surgery, cabergoline was restarted and increased to .5 mg twice daily.

Six months after surgery, the patient developed a new-onset severe headache and complete ptosis of the right eyelid with minimally reactive pupils in keeping with a right cranial nerve III palsy. His prolactin increased again to 816 mcg/L (816 ng/mL) as did the size of the tumor within the sella (1.4 × .9 × 1.3cm, previously .9 × .8 × .5cm; Fig. 4B). This prompted a second endoscopic transsphenoidal resection with postoperative fractionated radiation (54 Gy over 30 cycles) to the sella. Pathology remained unchanged from his first surgery. Prolactin declined to 172 mcg/L (172 ng/mL) on cabergoline .5 mg twice daily. Postoperative MRI of the sella showed distortion of the pituitary fossa, possibly related to treatment effect vs residual tumor (Fig. 4C).

One year after his second transsphenoidal resection, he then experienced worsening back pain, lower extremity weakness, and urinary retention. MRI of the brain and spine revealed numerous new lesions in the epidural space extending from the thoracic to sacral spine, with leptomeningeal enhancement filling the thecal sac at L5/S1 and extending to S3 (Fig. 4D). There was also a new suspicious 5.5 mm nodule inferior to the cerebellar tonsil (Fig. 4E and 4F). Serum prolactin increased to >4699 mcg/L (>4699 ng/mL). He received urgent lumbar decompression laminectomy and removal of the epidural tumor at L2-L3. Pathology confirmed metastasis, with histomorphology and proliferative indices unchanged compared to his first transsphenoidal resection.

## Outcome and Follow-up

Given his aggressive disease with tumor growth despite standard therapy, alongside cabergoline 1 mg twice weekly, he received 12 cycles of adjuvant monthly temozolomide (150-200 mg/m^2^). Initially, there was clinical and biochemical improvement with resolution of the 5.5 mm nodule, reduced epidural and leptomeningeal enhancement, and normalized prolactin to 9 mcg/L (9 ng/mL). However, during cycle 12, he was found to have a new nodule in the T3-T4 neural foramen and enhancement in the basal cisterns. He received stereotactic radiotherapy and resumed temozolomide chemotherapy along with capecitabine (750 mg/m^2^) for 3 cycles (Fig. 4G). He remained incontinent of bowel and bladder and immobile with severe leg weakness and unfortunately passed away 62 months from the time of initial diagnosis.

## Discussion

Hyperprolactinemia presents in .4% to 5% of the general population (3). Among its causes are prolactinomas, which have an estimated prevalence of 100 per million adults (4). Typically, patients with prolactinoma present with hypogonadism, low libido, and infertility (3). Men may experience erectile dysfunction and gynecomastia, while premenopausal women may present with menstrual irregularities, osteopenia, and galactorrhea (3). Generally, macroprolactinomas are associated with serum prolactin levels above 200 mcg/L (3).

Dopaminergics are the first-line therapy for the treatment of micro- and macroprolactinomas. Most patients respond within 6 months (5). Transsphenoidal surgery is second line, offered to symptomatic patients who cannot tolerate or who are unresponsive to dopaminergic agents.

Rarely, presumed prolactinomas can be prolactin-secreting pituitary carcinomas (6). Several clinical features may help to differentiate a prolactinoma from a prolactin-secreting pituitary carcinoma. Aggressive pituitary tumors tend to grow despite standard treatment, whereas most prolactinomas respond to dopaminergics within 6 months (1, 5, 7). Patients with prolactin-secreting carcinomas are more often male (3.7:1 male to female ratio) and over 50 years of age at diagnosis (6). Benign prolactinomas occur most commonly in females between the ages of 20 and 50 (6). Aggressive pituitary tumors are usually macro-tumors at clinical presentation, though tumor size alone does not predict aggressive behavior (2, 7). Tumor cells displaying a high Ki-67 proliferation index may also indicate aggressive growth and poor prognosis (1, 8).

Our patient illustrated many features of an aggressive pituitary tumor. Importantly, despite first-line treatment with cabergoline, his prolactin level did not decline and, in fact, increased without improvement in clinical symptoms. Pituitary pathology demonstrated an atypically high Ki-67 proliferation index of 20% to 25%. He also experienced disease progression shortly after surgery, further reflective of aggressive tumor behaviour.

There have been other published cases of prolactin-secreting pituitary carcinomas with metastasis to the spine, leptomeninges, and/or thecal sac (2, 9-13). The majority presented as macroadenomas (2, 9-13). The latency period between patient presentation and diagnosis of carcinoma ranged between 0 and 32 years (2, 9-13). The clinical course of pituitary carcinoma has been variably described. Some patients have had multiple recurrences and early metastasis (7, 9, 12, 14) and others, a protracted course of quiescence followed by rapid tumor growth (7, 10, 11, 13, 14). Time to diagnosis of metastasis is also variable. Indeed, in 1 case, the diagnosis of carcinoma was made 32 years after initial presentation (13). Other patients have had a short time to diagnosis, including our patient, as well as a 32-year-old female from Turkey who presented with metastases on initial assessment (12) and a 54-year-old female from the United States diagnosed with metastases shortly after transsphenoidal resection (2). Earlier medical care and aggressive therapy may contribute to early diagnosis of this condition.

Our patient's Ki-67 proliferation index was higher than in other reports (9-11). The World Health Organization formerly suggested that a Ki-67 index greater than 3%, elevated mitotic index, and extensive staining for p53 might predict poor prognosis and that adenomas with these features might be labeled as “atypical” (8). However, given that the prognostic significance of these criteria remain uncertain, they were not included in the updated 2017 guidelines (8). Unfortunately, there are no histological features to fully distinguish pituitary carcinoma from prolactinoma (8). We do suggest that a high Ki-67 value retains utility in identifying which patients should be watched more closely.

Radiotherapy is considered for pituitary carcinoma when there is tumor growth despite medical treatment and transsphenoidal surgery (5). Radiation consists of either external beam radiation therapy (EBRT) delivered bitemporally or bitemporally and frontally in fractionated doses. Stereotactic radiosurgery (SRS) provides the total radiation dose in a single session using a precise stereotactic frame (15). Generally, SRS is favored over EBRT due to convenience and safety, although it has limitations in the management of patients with diffusely infiltrating tumors and/or where the disease is too close to the optic chiasm (16).

There is controversy surrounding the potential pathogenic role of radiotherapy in the transformation of a benign pituitary tumor into malignant disease (17). In a retrospective study of 4905 patients presenting with benign tumors and treated with SRS (641 pituitary tumors), only 2 tumors over 8 years were suspected to have transformed, with a risk that was not statistically different from the risk of developing a malignant central nervous system tumor in the general population (18). Another retrospective study of 8917 patients (6750 pituitary tumors) found that although both EBRT and SRS were associated with an increased incidence of subsequent malignant brain tumors; tumors were unrelated to the pituitary (19). Both EBRT and SRS have, however, been shown to decrease prolactinoma tumor volume and prolactin levels, and nonrandomized studies of radiotherapy in association with temozolomide chemotherapy demonstrated superior tumor control over either temozolomide or radiotherapy alone (16).

Our patient was also treated with temozolomide, and a 2017 systematic review of case reports found that 76% (of 42 patients) who had resistant prolactin-secreting pituitary adenomas or carcinomas who received 1 to 24 cycles of temozolomide exhibited tumor shrinkage (20). Pathology displaying low MGMT staining, as in our patient, was also associated with a good response to this therapy (20). This stain is available at most neuropathology centers and may help guide further therapy. The 2018 European Society of Endocrinology guidelines suggest temozolomide therapy as first-line chemotherapy for aggressive pituitary tumors and carcinomas following documented tumor growth despite optimal standard therapies (7).

Aggressive pituitary tumors often, but not always, display high Ki-67 index, elevated mitotic index, and/or p53 staining on pathology, and therefore recommending temozolomide therapy based upon pathology results alone is not currently recommended (7). The European Society of Endocrinology guidelines suggest continuing temozolomide for at least 6 months or longer if effective (7, 15). However, whether a longer treatment duration of temozolomide in responsive patients improves the chance of continued remission is unclear; our patient's pituitary carcinoma recurred during cycle 12 (7). Moreover, the extent to which the combination of temozolomide with capecitabine is superior over monotherapy in controlling pituitary carcinomas remains to be determined (7).

## Learning Points

- Pituitary carcinomas are rare. However, being attuned to this diagnosis remains important due to its poor prognosis.
- Features that suggest pituitary carcinoma include its diagnosis in male patients above age 50 and a high Ki-67 index on pathology. The majority of aggressive lesions are macro-tumors at presentation. Elevated prolactin and tumor progression on standard treatment, including dopaminergic agents, surgery, and radiotherapy, also suggest aggressive tumor behavior.
- Tumors displaying low MGMT staining may have a favorable initial response to temozolomide chemotherapy, though it is unclear whether longer treatment duration in responsive patients enhances durability of this response.

## Data Availability

Original data generated and analyzed for this case report are included in this published article.
